# The Injury/Illness Performance Project (IIPP): A Novel Epidemiological Approach for Recording the Consequences of Sports Injuries and Illnesses

**DOI:** 10.1155/2013/523974

**Published:** 2013-11-27

**Authors:** Debbie Palmer-Green, Colin Fuller, Rod Jaques, Glenn Hunter

**Affiliations:** ^1^University of Nottingham, Nottingham, UK; ^2^F-MARC, Zurich, Switzerland; ^3^English Institute of Sport, Bath, UK; ^4^UK Sport, London, UK

## Abstract

*Background*. Describing the frequency, severity, and causes of sports injuries and illnesses reliably is important for quantifying the risk to athletes and providing direction for prevention initiatives. *Methods*. Time-loss and/or medical-attention definitions have long been used in sports injury/illness epidemiology research, but the limitations to these definitions mean that some events are incorrectly classified or omitted completely, where athletes continue to train and compete at high levels but experience restrictions in their performance. Introducing a graded definition of performance-restriction may provide a solution to this issue. *Results*. Results from the Great Britain injury/illness performance project (IIPP) are presented using a performance-restriction adaptation of the accepted surveillance consensus methodologies. The IIPP involved 322 Olympic athletes (males: 172; female: 150) from 10 Great Britain Olympic sports between September 2009 and August 2012. Of all injuries (*n* = 565), 216 were classified as causing time-loss, 346 as causing performance-restriction, and 3 were unclassified. For athlete illnesses (*n* = 378), the majority (*P* < 0.01) resulted in time-loss (270) compared with performance-restriction (101) (7 unclassified). *Conclusions*. Successful implementation of prevention strategies relies on the correct characterisation of injury/illness risk factors. Including a performance-restriction classification could provide a deeper understanding of injuries/illnesses and better informed prevention initiatives.

## 1. Introduction

Recognition of the importance of injury and illness epidemiology research has grown in the last 10 years with international governing bodies of sport and the International Olympic Committee (IOC) regularly conducting surveillance studies at major sporting events [1–10]. The prevention of injuries and illnesses and the long-term protection of athletes' health are key factors influencing this growing interest [11]. In addition to the impact on an athlete's health, injuries and illnesses also impact on the athletes' ability to train and perform; these interruptions may affect their preparations for and availability to take part in competitions, which in turn impacts on their ability to achieve lifetime dreams and aspirations of sporting success. Information about the incidence, severity, and nature (location and type) of sports injuries and illnesses are all important, as together they quantify the overall risk of injury and illness to athletes and thus provide information that allows the prevention initiatives to be correctly prioritized [12].

The ability to describe the incidence, nature, and causes of injuries and illnesses reliably has been recognised through the development and publication of injury surveillance consensus statements [13–18]. The consensus statement for football defines an injury as “any physical complaint sustained by a player…irrespective of the need for medical-attention or time-loss from activities” [13], while for Olympic sports an injury/illness is defined as “any musculoskeletal complaint…that received medical attention regardless of the consequence with respect to absence from competition or training” [14]. Once the injury definition is set, subclassifications by consequence are used to determine what becomes a recordable event, such as (but not limited to) whether the injury/illness results in “tissue damage”, “medical-attention” (without time-loss), “time-loss”, and/or “missed match/competition”.

Limitations with current consensus statements have been recognised for several years. For example, Fuller et al. [19] discussed how injury recurrences, reinjuries, and exacerbations could be recorded; Bahr [20] discussed how overuse injuries often limited athletic performance but were not recorded in injury surveillance systems if there was no time-loss; and Creighton et al. [21], discussed how injury severity was dependent on whether return-to-play decisions were based on an athlete's full return to sport without limitation or were based on limited return to training. Some injury surveillance studies have addressed the issue of continued participation or return-to-play under restricted performance conditions. Palmer-Green et al. [22] presented a methodology for recording impaired athlete availability and performance due to injury and illness in several sports during Great Britain Olympic team preparations for the Summer and Winter Olympics; Clarsen et al. [23] reported a methodology for recording over-use injuries of specific anatomical areas that resulted in restricted function of performance; and Jacobsson et al. presented a methodology for recording alterations in normal training due to the injury in athletics [24].

Most surveillance studies have focused on the etiology of “medical-attention” and/or “time-loss” definition of injury and illness incidents, but few epidemiological studies have related these events to an athlete's consequential training limitations. Regular training enables athletes to gain various sport-specific positive adaptations in skill development and physiological parameters designed to improve their performance [25]. Cessation or a marked reduction of the training stimulus can lead to a partial or complete reversal of a number of cardiovascular and musculoskeletal adaptations, [26, 27] and these reversals will ultimately compromise athletic performance. The magnitude of the detraining characteristics caused by injury/illness depends on the duration of training cessation (complete time-loss) and/or the duration of limited training (restriction) [28, 29]. The issue of impaired training and competitive performance, however, is not limited to overuse injuries, as limitations can also be associated with illness and acute, traumatic, gradual onset, and chronic injuries. The aim of this paper is to describe in detail (with illustrative data examples) an epidemiological recording system method based on the impact of injury/illness events on an athlete's capability to train/compete, previously reported briefly at the IOC World Conference of Prevention of Injury and Illness in Sport in Monaco in 2011 [22].

## 2. Methods

The following sections outline the background of the methodology developed and implemented in the summer of 2009 to monitor the injuries and illnesses sustained by Great Britain Olympic athletes in the period leading up to the 2010 Winter and 2012 Summer Olympics and which was used to quantify the impact of injury/illness on athletes' training and competition.

### 2.1. Injury/Illness Status (Time-Loss or Performance-Restriction?)

**Figure 1:**
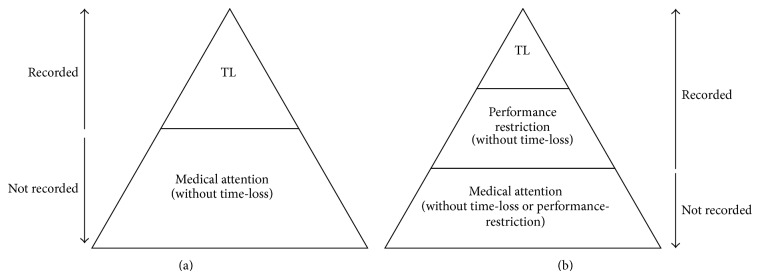
(a) Traditional hierarchy of injury/illness definition classification. (b) Alternative hierarchy of injury/illness definition classification. TL = time-loss; ∗ classification of an injury or illness is based on the initial status of the injury and not on any subsequent change of status during the time course of its recovery and ∗∗ while injuries/illnesses are always required to be classified as either time-loss or medical-attention (or performance-restriction) is should be noted that there can and always will be overlap between categories.

While the majority of sports injury definitions encompass a time-loss classification the definitions are somewhat categorical in their use of the terms ‘time-loss' or ‘absence' from training or competition that is complete absence. Limitations attached to these definitions have been described by Bahr; [20] in particular, it was questioned as to how appropriate these definitions were when applied to sports such as swimming or athletics where there are few time-loss injuries but an abundance of overuse/chronic performance-limiting injuries [4, 5, 8]. Bahr [20] focussed his discussion on the causes of performance-limiting overuse injuries but, because many athletes continue to train (and even compete) at high levels when experiencing pain and loss of function through injury or illness [20, 23, 30], he also highlighted the need to develop scales for assessing function-limiting injuries. Subsequent papers began to propose alternate methods in injury recording around specific joints, overuse injury, and athletes' levels of impairment [23, 24]. The focus of the present study was to develop a methodology that provided a measure of the impact of both time-loss injuries/illnesses and injuries/illnesses that did not meet the definition of “time-loss” but resulted in a restriction or modification to an athlete's regular training (e.g., an upper respiratory tract illness lasting 20 days resulting in a 60% level of training capacity). If this latter type of illness was included as a recordable event according to most consensus methodologies, it would create an overestimation of the illness burden (because 20 full days of training were not lost). Conversely, if the strict “absence” criteria were applied, the event would not be recorded despite resulting in a significant impact on the athlete (i.e., 40% reduction in training capacity over a period of 20 days). The division between medical-attention (with no time-loss) and time-loss definitions is therefore blurred, with the potential for some injury/illness events to lie between the two extremes. Figures 1(a) and 1(b) provide a visual description of this situation with Figure 1(b) presenting a potential alternative hierarchy.

### 2.2. Time-Loss and Performance-Restriction Injuries/Illnesses and Restriction-Impact

Time-loss injuries/illnesses are regarded as those events resulting in 100% restriction (i.e., complete time-loss), with the severity of these injuries/illnesses measured from the date of onset of the injury/illness (DOI) to the date of return to “full fitness” (DOR). DOI was defined as the date of sudden onset, or, for gradual onset injuries/illnesses, the date the athlete was first unable to take a full part in training or competition; full fitness was defined as the athlete being able to complete his/her full and normal training/competition. Performance-restriction injuries/illnesses are those events that result in an athlete only being capable of a reduced level of performance (1 to 99% restriction). The severity of these injuries/illnesses is calculated using two parameters: (1) number of performance-restricted days of training/competition in the period between DOI and DOR and (2) percentage of performance-restriction in training/competition during the performance-restricted days. The percentage of performance-restriction (i.e., {100 − level  of  performance}) denotes the percentage of training/competition restriction compared to the athlete's normal training regimen each day/week. This parameter is meant to provide performance rather than clinical relevance and is a function of the restriction to both the athletes training volume and training intensity, with equal weighting given to decrements in each; for simplicity, percentage scores were denoted by 5% increments for medical staff to select on the report form (after discussion with the athlete/coach on the level). The percentage performance-restriction value multiplied by the duration of restricted performance provides a quantified measure of the impact of the injury/illness on the athletes performance, that is, the restriction-impact. As an example of the utility of this approach, two restriction injuries both lasting 10 days may appear to impact equally on an athlete; however, if the percentage of performance-restriction in the first case was mild (10%) and in the second case moderate (50%), the restriction-impacts of the two events would be full-time equivalent (FTE) severities of 1 day and 5 days, respectively, which reflects the greater impact of the latter injury on the athlete. All results for performance-restriction injury/illness severity are presented as restriction-impact full (FTE) days, in line with the traditional way of reporting time-loss days.

### 2.3. Changes in an Athlete's Injury/Illness Status

**Figure 2:**
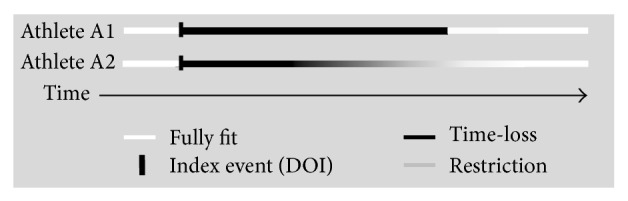
Traditional time-loss definition compared to the inclusion of a performance-restriction injury/illness classification and change of injury/illness status.

A 60-day time-loss injury implies that the athlete had 60 days absence from training/competition and returned to full training on day 61 (Figure 2, Athlete A1). In reality, there is more likely to be a graded return to normal full training comprised of increases in training intensity and volume (Athlete A2); in terms of athlete performance, the 60 days may therefore be an overestimation of the true impact of the injury/illness. Bahr [20] described the potential complexity of the time-line for an overuse injury and recommended that continuous or serial measurements should be made on an athlete's condition in order to monitor these changes; the situation is similar for all injuries and illnesses, including acute and chronic events. Hence, the proposal here is that any change of status (COS) in an athletes' ability to train/compete over time is recorded. This approach is particularly beneficial when tracking complex long-term injuries/illnesses, as it provides sensitive, real-time capture of information about the evolution of and recovery from an injury/illness. The date of change of status is simply taken when the athlete moves from complete time-loss to performance-restriction or vice versa. The total severity of an injury/illness is then calculated by summing the individual time-loss and restriction-impact (FTE) days.

**Figure 3:**
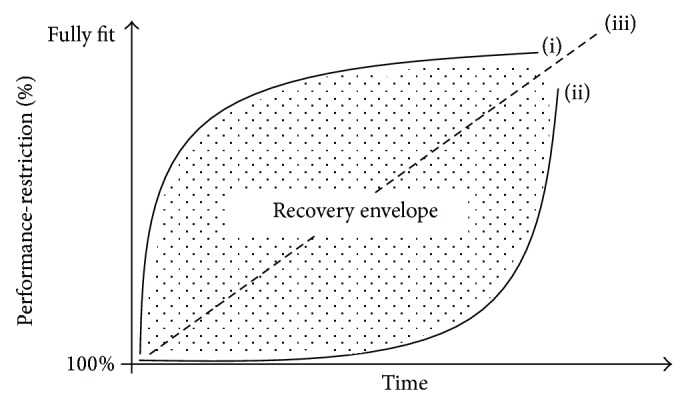
The performance-restriction injury/illness recovery envelope. (i) Fast/slow recovery; (ii) slow/fast recovery; and (iii) linear recovery.

The progression of an athlete's return to full fitness from a period of performance-restriction will depend on the level of performance-restriction at DOI and/or COS. Few injuries/illnesses display a truly linear return to fitness with time, and Figure 3 represents a conceptual schematic of this recovery relationship. The performance-restriction recovery envelope can be used to compare the recovery of an athlete experiencing (i) a rapid early progression followed by a final slower period of progression with an athlete experiencing (ii) a slow early progression followed by a rapid end-phase recovery to full fitness.

### 2.4. Athlete Pain Score

All athletes experience pain during their careers, whether through the normal rigors of training or as the result of injury [31]. Athletes often train and compete with injury/illness-related pain but how frequently this occurs and what the impact is on an athlete's ability to continue normal training are largely unknown. In two recent studies, it was reported that 52% of NFL players [32] and 60% of players at the 2010 FIFA football World Cup [33] used prescription pain killers during play. Although it has been recommended that surveillance methods should include the measurement of pain alongside days of absence [20], there is little other evidence on the need for recording pain [31] or that pain is being recorded within longitudinal surveillance studies. When athlete pain has been recorded alongside the incidence of injury, it has only been done retrospectively rather than prospectively at the time of injury [34]. It is proposed, as a component of injury surveillance, that pain could be used as an indicator of injury severity. Although there are no published data currently available, it is reasonable to assume that, at their onset, severe acute injuries such as fractures may elicit higher levels of pain for an athlete than chronic injuries such as a tendinopathy.

**Figure 4:**
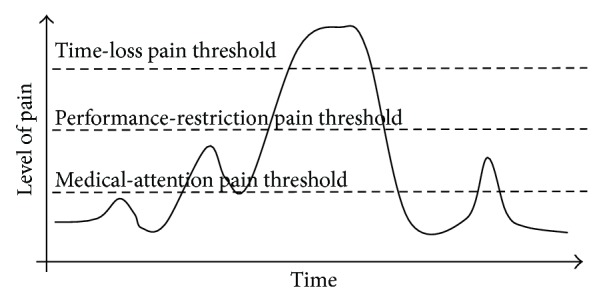
Theoretical model of pain and injury consequence (adapted from Bahr, [20]).

Multiple person-to-person physical and psychological factors and sport-to-sport sociological and environmental factors combine to determine an athlete's threshold and tolerance to pain, [31] and athletes may experience time-loss or restrictions to training/competition at different points along their own pain continuum.  Figure 4 is an adaptation to the pain model presented by Bahr [20] that shows injury pain thresholds for an athlete moving from medical-attention through performance-restriction, complete time-loss, and recovery.

### 2.5. The Great Britain Injury/Illness Performance Project (IIPP)

The current paper summarises the results recorded during the injury/illness surveillance study of Great Britain Olympic athletes conducted under the auspices of the UK Sport/English Institute of Sport Injury/Illness Performance Project (IIPP) and provides some illustrative example of the benefits of the proposed methodology. The IIPP methodology, which was based around the IOC, FIFA, and IRB injury/illness consensus statements, [13–15] included the methodological adaptations discussed above, and used the Sport Medicine Diagnostic Coding System [35]. The IIPP study involved 322 athletes (males: 172; female: 150) from 10 Great Britain Olympic sports (summer: 7; winter: 3), badminton (*n* = 21), canoe/kayak (*n* = 43), curling (*n* = 29), cycling (*n* = 61), football (women: *n* = 17), Judo (*n* = 35), modern pentathlon (*n* = 11), short track speed skating (*n* = 11), skeleton (*n* = 14), and swimming (*n* = 80). The study period covered September 2009 to August 2012, with data collected prospectively for each sport, each year, from the start of preseason training until the end of the competitive season (summer sports: September to August; winter sports: April to March). Medical staff working full time with centralised athletes recorded all the details of athlete injury/illness data, including full diagnosis, whenever a recordable event occurred. Standardised medical report forms were used with a questionnaire return compliance rate of 98%. Training and competition exposure data for all athletes were recorded on a weekly standardised report form by coaching and conditioning support staff but are not reported within the present paper.

The IIPP methodology defined any injury/illness that negatively impacted on an athlete's ability to undertake full and normal training as a recordable condition, irrespective of the athlete's ability to compete; “medical-attention” injuries/illnesses that did not impact on an athletes' ability to undertake full training/competition were not included. A recordable condition had two levels of subclassification: (i) time-loss, which was an injury/illness that caused an athlete's complete absence from all training/competition, and (ii) performance-restriction, which was an injury/illness where the athletes' training/competition continued but the volume and/or intensity were restricted or modified (e.g., a nonweight-bearing activity such as cycling replaced a weight-bearing activity such as running). The injury/illness initial status (i.e., time-loss or performance-restriction) was defined at the date of injury (DOI), irrespective of any subsequent change of status.

Severity data were reported in days for both time-loss and performance-restriction. Performance-restriction was reported as previously described as restriction-impact (FTE) days, with the number of performance-restricted days presented in superscript; for example, 18^33^ severity days means that the performance-restriction injury lasted for 33 days during which the level of restriction equated a restriction-impact of 18 full (FTE) days lost. Time-loss injuries that had a change of status to a period of performance-restriction also display the number of days the injury lasted for (time-loss days added to performance-restricted days).

Significant differences were calculated using two-tailed *Z* tests [36]. Significance was accepted at *P* ≤ 0.05 (equal variances assumed) and exact *P* values are reported throughout. Ethical approval for the study was obtained from the UK Sport (London) ethics advisory board.

## 3. Results

During the study, 565 injuries (competition: 98, training: 461, and unclassified: 6) and 378 illnesses were recorded. Of all injuries, 216 were classified as causing time-loss and 346 as causing performance-restriction (3 were not classified by medical staff at the time of recording) (Table 1). On average, each injury resulted in 15 days missed training (competition: 26 days; training: 12 days) and each illness 6 days, with time-loss injuries/illnesses more severe than performance-restriction injuries/illnesses. The numbers of time-loss (50) and performance-restriction (47) injuries occurring in competition were similar (*P* = 0.57); however, within training significantly more (*P* < 0.01) injuries resulted in performance-restriction (284) than time-loss (160). For athlete illnesses, the majority of events recorded (*P* < 0.01) resulted in complete time-loss (270) compared with performance-restriction (101) (7 unclassified).

**Figure 5:**
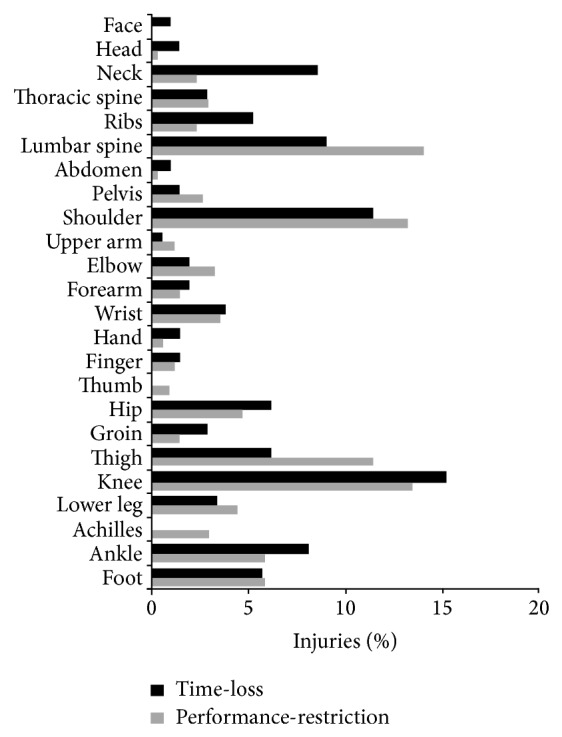
Percentage of injuries by location, as a function of injury status.

By injury cause, 53% of all injuries were classified as overuse (gradual/sudden onset) and 47% as acute (*P* = 0.19). The majority of overuse injuries (72%) resulted in performance-restriction, while, conversely, 59% of acute traumatic injuries resulted in complete time-loss. Injuries to the knee, shoulder, and lumbar spine were most common, with more knee injuries resulting in time-loss but conversely more shoulder and lumbar spine injuries resulting in performance-restriction (Figure 5).

**Figure 6:**
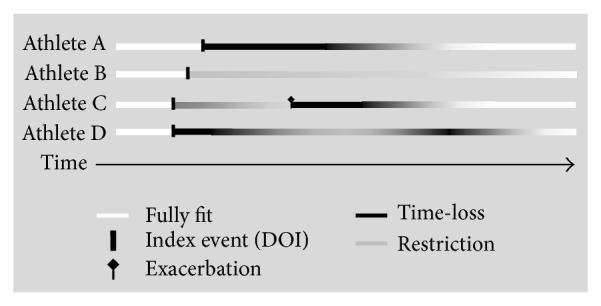
Examples of injury/illness occurrences collected during the IIPP based on time-loss and performance-restriction status.

Figure 6 provides four examples of time lines of injuries/illnesses recorded within the IIPP study. Athlete A sustained a lower leg fracture initially causing complete time-loss from all competition and training, followed by a prolonged period of graduated training back to full fitness. Athlete B had a shoulder tendinopathy injury, which did not result in time-loss but impacted over an extended period of time on aspects of his training until his return to full fitness. Athlete C initially suffered a recurrence of a chronic lumbar spine disc injury causing the athlete to restrict her training; the athlete's condition began improving but there was a sudden exacerbation of the injury causing complete time-loss. As her condition improved once again, this athlete returned to restricted training where loads (intensity and volume) were progressively increased until she returned to full fitness. Athlete D presented a chronic fatigue illness with varying periods of complete time-loss and restricted training before returning to full fitness.

**Figure 7:**
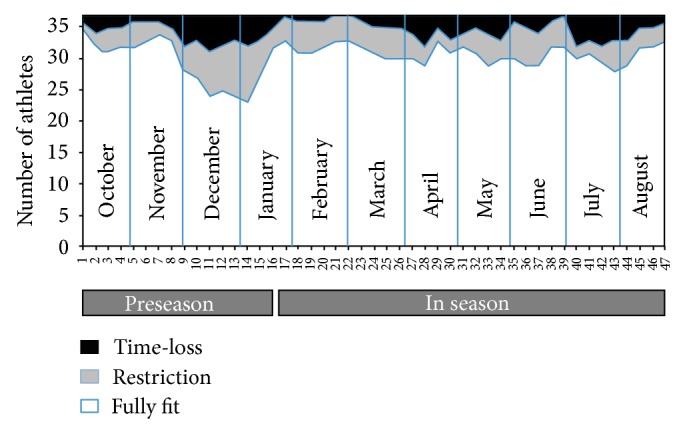
Great Britain sport (water-based) squad status 2010/11.

The IIPP study methodology provided Great Britain sports with a summary of athletes' ongoing time-loss and performance-restriction and the squad availability for training/competition.  Figure 7 presents a sport's (water-based) squad data during the 2010/11 season; on average at any time during the season, 18% of the team were unavailable for full training/competition, with December and January posing the period of greatest impact on training as a consequence of injury or illness.

### 3.1. Pain

**Figure 8:**
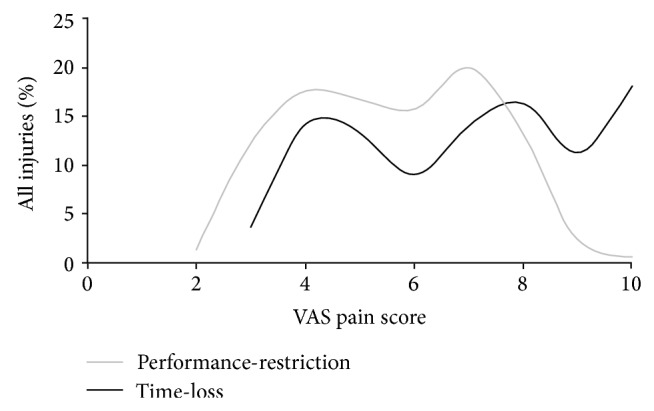
IIPP VAS pain score (at DOI) distribution for time-loss and performance-restriction injuries recorded between 2009 and 2012.

For all injuries reported in the IIPP study, athletes were asked to rate their pain at the initial date of injury (DOI) using a visual analogue scale (VAS) from 0 (no pain) to 10 (the worst pain). The athlete's pain scores were also recorded at each COS and DOR. All injured athletes reported having some level of pain at DOI (VAS: mean = 6.1; SD 2.1) and 39% some level of pain at DOR (VAS: mean = 1.8; SD 1.0).  Figure 8 shows the distribution of pain levels at DOI for time-loss and performance-restriction injuries, with pain scores generally higher for time-loss compared with performance-restriction injuries. With the majority of time-loss injuries reported to be acute in nature and performance-restriction injuries to be overuse, this supports the theory proposed that acute injuries present more pain.

## 4. Discussion

When dealing with elite sport, the consequence of an athlete's injury/illness is often dependent on the sport involved. Injury and illness events may be of less consequence within team sports (because substitutes are normally used), some individual sports, where the format of competition is such that performance may be measured over an extended period of time (e.g., a season), and/or during particular times of the year that is preseason compared with in season. However, for athletes competing in many individual sports, the focus is on achieving peak performance at possibly one major event each year, for example, World Championship or Olympic Games. Injury/illness events are likely therefore to have a bigger influence on athletes competing in individual sports as these athletes must attain optimal conditioning and perform maximally at specific times, when winning margins are often measured in fractions of a second or millimeters.

Successful implementation of injury and illness prevention strategies relies on the correct characterisation of injury/illness risk factors, [37] and accurately assessing the consequences and severity of all sports injuries/illnesses is a crucial aspect in identifying the extent of the injury/illness problem. Hence, the addition of the proposed performance-restriction definition, alongside the traditional time-loss definition, provides an important additional layer of information that was previously unavailable. In the IIPP study, using the performance-restriction injury classification, an additional 346 injuries and 101 illnesses were recorded, equating to just over twice as many injuries and a quarter more illnesses reported compared to the traditional time-loss definition. This was similar to the findings by Clarsen et al. [23], who also reported that significantly more overuse injuries were recordable using a new overuse injury questionnaire compared with standard time-loss injury registration methods. In a subsequent paper, the same authors proposed adaptations to their overuse injury method to include the recording of all injuries and illnesses, again with similar more detailed results [38].

In addition to capturing more impactful injury/illness issues, the IIPP methodology allowed the use of standard days lost for reporting severity of performance-restriction so that data could be comparable with a time-loss definition. This provided more accurate information on the impact of injury and illness issues whereby potential overestimations were negated in terms of full-time equivalent days lost from training/competition compared with a pure time-loss definition. The use of full-time medical practitioners to record medical data including specific diagnoses also avoided reliability and validity issues related to athlete self-reporting.

For prevention initiatives to be effective, translation and implementation of research findings in a real-world setting are paramount [39]. Hence, full NGB/sporting body engagement is important not only for data returns compliance but also in provision and feedback of injury/illness data, so that information provided is acted upon and strategies adhered to. While not quantified in the present study currently, it is known that targeted prevention initiatives based on recommendations from biannual reports (and ad hoc NGB requested analyses) are being implemented by IIPP sports NGB's with positive reductions in days lost to illness and injury. This was particularly evident in the run up to the London 2012 Olympic Games.

The inclusion of a performance-restriction classification also raises the possibility of examining the efficacy of current treatment and rehabilitation algorithms for injured/ill athletes from a different perspective. For example, is it better to allow an athlete to continue training at a restricted level, or should the athlete experience a complete break from training? It is reported that some of the negative effects of missed training can be avoided or limited by using reduced training strategies (e.g., reduced training volume), as long as the training intensity is maintained and the frequency of exposure is only reduced moderately [28]. Cross-training or a modified-training mode may also be beneficial in maintaining training-induced adaptations during periods of injury/illness [29].

Recording pain scores alongside performance-restriction injuries/illnesses will help provide a better description of those situations discussed by Bahr where athletes experience ongoing pain but do not sustain a time-loss injury [20]. The preliminary results presented here demonstrate the high proportion of athletes returning from injury to training/competition while still experiencing residual levels of pain; this supports the recent findings among NFL and football players where a large proportion of players were competing in pain [32, 33]. This type of information could be of great importance for studies of the consequences of long-term injuries experienced by many athletes (e.g., osteoarthritis). In addition, the distributions of pain scores for both time-loss and performance-restriction injuries in the current study show distinct bimodal responses. These distributions could arise for many reasons, the most common being that the distribution is made up of two unimodal distributions. If this was the case, it would suggest that there could be two different groups of athletes in terms of reporting or perceptions of pain, one with moderate (VAS: 3.0 to 5.0) and one with a higher (VAS: 6.5 to 8.5) level of pain. Conversely, this could represent the level of pain for two distinct groups of injuries, that is, the distribution of overuse injuries (the first peak) and acute injuries (the second peak). How the body reacts to pain is a complex process, [31] and the proposals for recording pain in epidemiological studies of sports injuries could provide opportunities to explore new perspectives in this area.

It is unlikely that a single universal system will be suitable for the needs of injury/illness studies in all populations and settings [40, 41], and one of the limitations of the proposed methodology is the additional time required of the medical staff to record the performance-restriction and pain score information. Therefore, the increased level of data collection proposed might not be appropriate for studies with large populations, for some contact sports where the incidences of injury are high and where full-time medical staff may not be available to collect the information. In addition, while clearly defined criteria are established for “medical-attention,” “performance-restriction,” and “time-loss” definition classifications, it is recognised that these thresholds may also still be blurred in real-world settings, for example, “medical-attention” classified injuries/illnesses may result in some restriction to training/competition but these restrictions may go unreported.

In the IIPP study, semiquantitative measures of training intensity and volume were used. Although research suggests that greater weight should be attached to a reduction in intensity due to greater detriments to subsequent performance, [29] for simplicity and ease of practitioner use, the present paper proposed equal weighting be given to both intensity and volumes. It would be preferable in future studies of this type if quantified measures of intensity and volume (and restrictions there-of) could be used (e.g., intensity linked to heart rate or Vo_2_ max data; volume linked to GPS data) to provide more accurate values (although this may not be appropriate for some pure skill-based sports). An additional parameter, which measured the effectiveness of changes in training modality, such as replacing weight-bearing with nonweight-bearing activities, could also be added to provide more information in some studies.

## 5. Conclusion

Although training methods seek to improve athlete performance through the aggregation of marginal gains [42], the impact of injuries/illnesses on training is a key perspective, particularly in an elite sport setting. Where previously, time-loss has been used as a gross measure of the consequence of injuries/illnesses in sport, new discussions are emerging on the merits of alternate methods for quantifying levels of injury/illness-related athlete function and restriction in training. The current proposal to include an additional category of performance-restriction and pain scores, could provide greater detail in the understanding of the impact of injuries and illnesses on athlete availability and performance. With better understanding of this relationship, prevention initiatives can be better targeted to reduce the impact of athlete injuries and illnesses and improve athletic performance. The IIPP study continues into its fifth year of data collection in Great Britain Olympic athletes preparing for the Sochi 2014 and Rio 2016 Olympic Games.

## Figures and Tables

**Table 1:** Injury and illness number (and severity mean days^lasted-for^in parenthesis), by status.

	Performance-restriction	Time-loss	Unclassified	Total
Competition	47 (18^33^)	50 (35^54^)	1 (11)	98 (26^44^)
Training	297∗∗ (9^25^)	163 (18^30^)	1 (14)	461 (12^27^)
Unclassified	2 (21^62^)	3 (55^87^)	1 (1)	6 (28^66^)
All injury	346 (10^26^)	216 (23^36^)	3 (9)	565 (15^31^)
All illness	101∗∗ (5^11^)	270 (7^8^)	7 (4^9^)	378 (6^9^)

^**^ *P *≤ 0.01: significant difference between number of time-loss and performance-restriction injuries/illnesses.
